# Exceptional Presentation of Heart Thrombi Secondary to Myocardial Infarction Due to COVID-19 Pandemic

**DOI:** 10.7759/cureus.17926

**Published:** 2021-09-13

**Authors:** Julio A Ovalle Ramos, Munira M Sirajum

**Affiliations:** 1 Internal Medicine, Lincoln Medical and Mental Health Center, Bronx, USA; 2 Cardiology, Lincoln Medical and Mental Health Center, Bronx, USA

**Keywords:** left ventricular thrombi, myocardial infarction, covid and heart, coronavirus pandemic, myocardial infarction complication

## Abstract

Left ventricular thrombi (LVT) is an uncommon complication that can occur after a myocardial infarction thanks to the discovery of revascularization therapies. Before it, an LVT was described in up to 60% of patients with myocardial infarction. The authors present a case of a 46-year-old female who presented to the emergency department with one week of dyspnea, who had symptoms of chest pain for a week, however, did not show up in the hospital due to the ongoing COVID-19 pandemic. In-patient new-onset heart failure workup during that time was minimized due to the state of emergency COVID-19 pandemic. The patient lost to follow up appointment and then presented again to the hospital with Echocardiogram at that time showing mid to distal septal and apical hypokinesis, EF 30%-35% and a highly mobile circumferential echogenic mass of 2.4x2.4 cm noted in the left ventricle (LV) with differentials of LV thrombus vs cardiac tumor. Hospital complicated by LV thrombus embolization with bilateral lower extremities (LEs) arterial thrombi and limb ischemia. Left cardiac cath with a result of severe triple vessel disease requires either coronary artery bypass grafting (CABG) or complex percutaneous coronary intervention (PCI). A tentative plan was to pursue CABG, however, lower extremities must be healed prior to cardiothoracic surgery.

## Introduction

Myocardial infarction (MI) has been linked to many cardiovascular complications. Due to the regional wall abnormalities after the event, there is an increased risk of left ventricular thrombi (LVT) that can be seen 3-10 days post-MI. LVT is uncommon in patients post-MI with early revascularization but in the pre-thrombolytic era was described in up to 60% of patients with anterior MI [1]. The aim of this case is to show the clinical impact of the loss of follow-up due to the COVID-19 pandemic and its effects on the increase in the presentation of complications of LVT post-ST-elevation MI (STEMI) in the era of reperfusion therapy. It also suggests this complication is underestimated due to late presentation post STEMI during a pandemic period.

In the new era of percutaneous coronary intervention (PCI), the incidence of LVT as a complication of STEMI is not well established but has been a debate for the past four decades [2]. Previous studies [3] have shown that LVT is more frequent in acute anterior or apical myocardial infarction, demonstrating a 0.3% incidence in non-anterior MI. It is also stated that 5%-15% of patients that developed STEMI are still predicted to have an increased possibility to developed LVT for up to 3-6 months [4].

## Case presentation

**Figure 1:**
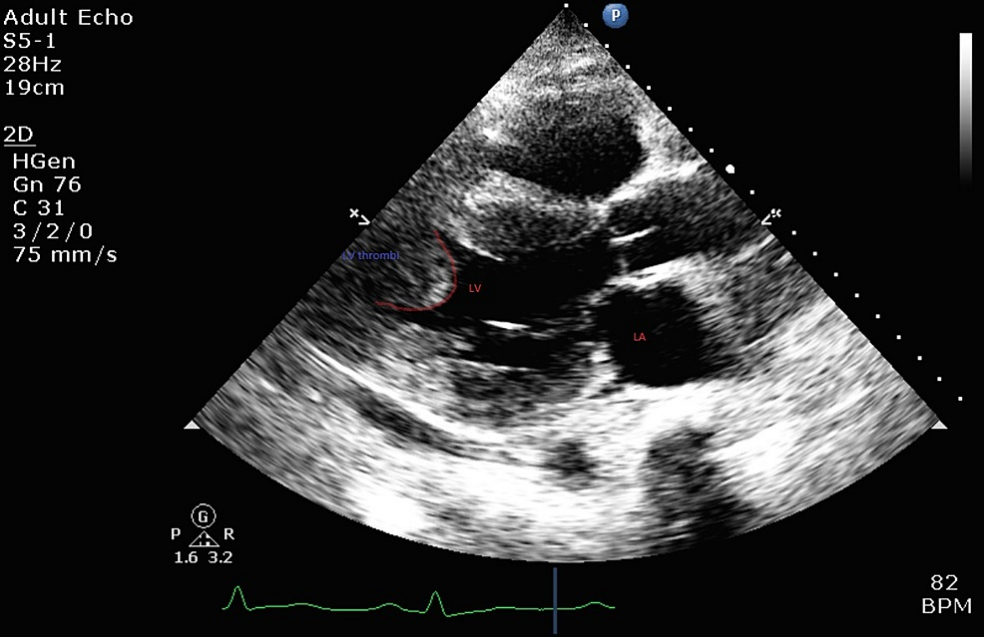
Echogenic mass of 2.4x2.4 cm noted in the left ventricle

A 46-year-old female presented to the emergency department with one week of dyspnea with past medical history included hypertension, dyslipidemia, uncontrolled type 2 diabetes mellitus, active smoker, morbid obesity, medication non-compliance and schizoaffective disorder. The patient had a previous admission three months ago with new-onset heart failure (HF) symptoms. Prior to that first admission, the patient had symptoms of chest pain for a week; however, she did not show up in the hospital due to the COVID-19 pandemic. At that time found to have an ejection fraction (EF) of 35% with diffuse wall hypokinesis seen in a multiple-gated acquisition scan (MUGA). ECG at that time was with poor R-wave progression with minimal troponin elevation. The patient was placed on carvedilol, Lisinopril, aspirin, furosemide and atorvastatin during hospitalization. Due to no signs or symptoms of an acute coronary syndrome (ACS) at the time, a workup for new-onset HF was referred to outpatient as In-patient work up was minimized considering that the hospital was in a state of emergency by reason of the COVID-19 pandemic. The patient lost an echocardiogram (ECHO) and cardiology appointment due to the outgoing COVID-19 pandemic. During second admission an ECHO showed mid to distal septal and apical hypokinesis, EF 30%-35% and a highly mobile circumferential echogenic mass of 2.4x2.4 cm noted in the left ventricle (LV) with differentials of LV thrombus vs cardiac tumor (see Figure 1, Video 1). The patient was started on heparin drip with a plan to get cardiac magnetic resonance imaging (cMRI). As there was no cMRI or cardiac catheterization laboratory at the facility, the patient was transferred to another institution for further workup.

**Video 1:** Echogenic mass of 2.4x2.4 cm noted in the left ventricle

The hospital course in the Coronary Care Unit (CCU) was complicated by LV thrombus embolization with bilateral lower extremities (LE) arterial thrombi and limb ischemia requiring right common femoral thrombectomy, bilateral LE fasciotomies. A repeat ECHO with a decrease in mass/thrombus size to 1.8 x 1.2, apical, septum and inferior wall hypokinesis (unchanged from prior). Left cardiac Cath with a result of severe triple vessel disease including complete occlusion of mid-right coronary artery (RCA), partial occlusion of mid-left anterior descending (LAD) artery, and circumflex arteries (LCx) requiring either coronary artery bypass grafting (CABG) or complex PCI. Cardiac MRI showed a scar of the inferior and apical territory, implying viability in LCx and proximal/mid LAD distribution, possible viability RCA. Cardiothoracic surgery and cardiology discussed the patient and provided a plan for moving forward, tentative plan was to pursue CABG; however, LE must be healed prior to cardiothoracic surgery. The patient was discharged to sub-acute rehab (SAR) with outpatient plastics follow-up for wound management.

## Discussion

In a meta-analysis published by Heerajnarain et al. [5], LVT was reported as 6.3% by cardiovascular magnetic resonance within one month of presentation of STEMI patients. In the same publication, the presence of LVT was increased among patients with anterior MI to 12% and when adjusting for patients with LVEF < 50% has been reported as 19.2%.

Phan et al. [6] showed there is a significant LV dysfunction among the risk factors that predispose to LVT formation and patients with LVEF ≤ 40% and anterior infarct location could benefit to undergo both a baseline and follow-up CMR study to detect later LV thrombus formation. Before the presentation to the ER, our patient was previously admitted to the hospital with MUGA showing EF of 35% and diffuse wall hypokinesis that might be a predictor for the development of LVT post-MI in this patient.

LVT as a complication of STEMI may lead to increased mortality due to systemic embolization [6]. The risk of systemic embolism after LVT was reported to be approximately 20% [4]. Our patient had bilateral LEs arterial thrombi and the ECHO showed a decrease in size in the LVT been that the likely source of the emboli. It should be noted that even though the incidence of LVT is probably underestimated in the era of PCI, the presence of LVT should be assessed in patients with clinical characteristics that are seen to increase the risk of its development and that may subsequently cause systemic embolization. Clinical impact with huge morbidity related to the loss of follow-up due to the COVID-19 pandemic and complications of LVT post-MI in the era of reperfusion therapy.

Clear recommendations on how to prevent the formation of LVT in high-risk patient remains a challenge. McCarthy et al. [7] stated that data on effectiveness and safety of oral anticoagulants in the setting of LVT as a complication of MI may be difficult to obtain as randomized clinical trials appear unlikely to occur as the incidence of LVT formation after MI has decrease do the PCI era. Regardless of the existing amount of information, both the American College of Cardiology Foundation (ACCF) and American Heart Association (ACC) guidelines recommended considering the use of anticoagulation in patients with STEMI with anterior apical akinesis or dyskinesis by ACCF and patients with ischemic stroke or TIA in the setting of acute anterior STEMI with anterior apical akinesis or dyskinesis by AHA to prevent the formation of LVT and subsequent embolization to prevent further complications [7-9].

Robinson et al. [10], in a multicenter retrospective study to compare outcomes associated with direct oral anticoagulants (DOAC) use and warfarin use for the treatment of LVT, concluded that in more than 500 patients they review, DOAC was associated with increased risk of stroke or systemic embolism compared to warfarin. They were limitations in the study that can make an argument that comparison cannot be made until a prospective study can be assessed. As of now, both the AHA and ACCF have recommended vitamin K antagonist as the preferred drug for anticoagulation for the treatment of LVT as a complication of MI [8,10].

## Conclusions

The case illustrates an uncommon complication after a MI such as LVT in a setting of loss of follow-up and to seek medical attention when needed. This case presentation should create awareness for people with increased risk of cardiovascular events, and who may subsequently develop LVT and systemic embolization. As per the AHA and ACCF, vitamin K antagonists may be considered by physicians as prevention of systemic embolization of LVT. Further prospective studies are needed to make clear statements for both prevention and treatment for LVT formation.
